# TRIO Method for Detection of Beta-Lactams, Sulfonamides, and Tetracyclines in Raw Commingled Cows' Milk: *AOAC *Performance Tested Method*^SM^ 121601*

**DOI:** 10.1093/jaoacint/qsaa034

**Published:** 2020-03-05

**Authors:** Robert S Salter, R J Markovsky, D W Douglas, S J Saul, A C Tran, D R Legg, J A Schwartz, D M Conaway, L W McRobbie, Emily Kalinowski, Mary Bulthaus

**Affiliations:** 1 Charm Sciences, Inc., 659 Andover St, Lawrence, MA 01843, USA; 2 Eurofins-DQCI, 5205 Quincy Street, Mounds View, MN 55112, USA

## Abstract

A qualitative 3 min one-step assay for detecting beta-lactam, sulfonamide, and tetracycline antibiotics was validated following milk screening test guidelines developed by FDA-CVM, AOAC-RI, and IDF. The validated 90% detection levels with 95% confidence were: penicillin G 2 part per billion (ppb); amoxicillin 4 ppb; ampicillin 9 ppb; ceftiofur plus metabolites 50 ppb; cloxacillin 9 ppb; cephapirin 15 ppb; sulfadimethoxine 8 ppb; sulfamethazine 9 ppb; chlortetracycline 34 ppb; oxytetracycline 53 ppb; and tetracycline 42 ppb. Detection levels were lower than U.S. and Canadian allowable limits for milk and were consistent with most European Maximum Residue Limits. Tests of raw commingled cows’ milk indicated a low positive error rate of <0.3% with no interferences demonstrated by 1.08 MM/mL somatic cells, Gram-positive or Gram-negative bacteria < 300 K/mL, freeze/thawing, or non-targeted drugs. Detection of incurred residues were similar to, or more sensitive to, fortified samples. Some cross reactivity across drug families occurred in interference studies and therefore initial positive samples should be confirmed with drug family specific screening methods. The National Conference of Interstate Milk Shipments approval as a bulk tank/tanker screening test was completed in three stages for each drug family, including a tetracycline confirmation procedure to target U.S. tolerance levels. Detection and robustness were found to be appropriate for multiple countries’ regulatory requirements for screening tests. The method development, validation, and approval was intended to diversify and increase the verification tools for the control of the major antibiotic drug families used in managing cows’ health and welfare.

## Scope of Method

Charm ROSA TRIO test for raw commingled milk is a lateral flow test in Charm Rapid One Step Assay (ROSA) format. It is a competitive multiplex immuno-receptor assay used for simultaneous detection of the antibiotic classes, β-lactam, sulfonamide, and tetracycline, at Canadian MRL and U.S. Tolerances/targets in raw commingled milk samples taken from farm tanks and tanker trucks. Canadian MRL and U.S. tolerances/target testing levels are equivalent except for the tetracycline class where the Canadian MRL is 100 ppb and the U.S. tolerance is 300 ppb. The TRIO test is qualitative with drug family specific identification. The method detects the broad class of antibiotics from each family and does not identify the specific compound within the family of antibiotics that is causing a positive result.

## Definitions

### (a) Standard Deviation of Repeatability (Applicable to Reading)


$$s_{r}=\sqrt{\frac{\sum_{i=1}^{n}{\left(X_{i}-\bar{X}\right)}^{2}}{n-1}}$$

### (b) Relative Standard Deviation of Repeatability (CV%, Applicable to Reading)


$${\text{RSD}}_{\text{r}}=\;\left[{\text{s}}_{\text{r}}/{\text{mean}}_{\text{cand}}\right]\text{ × 1}00$$

*Probability of Detection (POD)*—The proportion of positive analytical outcomes for a qualitative method for a given matrix at a given analyte level or concentration. POD is concentration dependent and expressed as a percentage POD = [(#positive/#replicates) × 100].

## Principle

**Figure 1.**
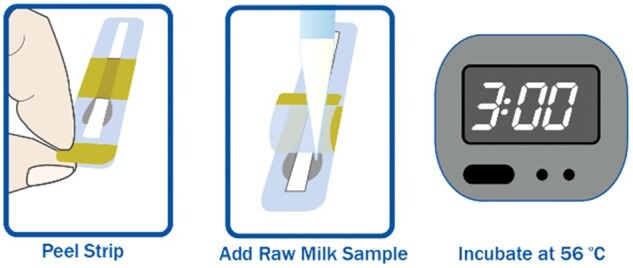
TRIO Method test flow chart and its possible line development interpretations. On the developed test strip there are four development lines, a control line (C) and three antibiotic specific test lines: BL = beta-lactam, S = sulfonamide, TE = tetracycline. When C is lighter than all three test lines the result is interpreted as Negative. When the particular test line is lighter than the control line the result is interpreted Positive for that line:-BL, -S, or -TE. When multiple test lines are lighter than C the Positive interpretation possibilities are: -BLS, -BLTE, -BLSTE, and -STE.

The TRIO test consists of a flow strip encased in a plastic outer container device. The flow strip consists of receptor and antibody binding agents attached to colloidal gold, a control line, and three detection lines, one for each drug class. The test lines are immobilized forms of the drug family where the BL line presents a β-lactam ring, the S Line a sulfonamide structure, and the TE line a tetracycline structure. Milk, 300 µL, is added to the sample compartment of the flow device placed in a 56°C incubator. As milk flows through the device, it hydrates the binding agents and any antibiotic in the milk sample will attach to the agents. As the binding agents flow across the detection lines, any unbound binding agent will attach and form a reddish BL, S, or TE line. Any antibiotic-bound binding agent will then be captured on the control (C) line. After 3 min the test lines are compared to the control line using a reader, model Charm-EZ, or Charm-EZ lite. If the test lines are the same or darker than the control line, then the sample is negative below the method’s level of detection. If any detection line is lighter than the control line, the sample is positive for a drug in the drug class of that binding line, BL (for β-lactam), S (for Sulfonamide), or TE (for Tetracycline). The reader has the option to tell which line is positive, BL, S, or TE or their combination if multiple lines are lighter than the control line. Any combination of multiple positive lines is possible, BL, S, TE, BLS, BLTE, STE, BLSTE, as shown in Figure 1. Alternatively the reader option can be turned off and only a positive will be delivered without drug line information.

## General Information

Antibiotic use in milk-producing animals is an important option for maintaining animal health and well-being (1). Antibiotic screening of raw milk before acceptance into processing plants is a prudent risk control that is regulated worldwide to prevent the unintended human consumption of antibiotics (2, 3). Antibiotic residues increase the risk for antimicrobial resistance, allergic health reactions, or potential economic damage to a dairy’s wholesome image (2). There is a broad variety of antibiotics to choose from based on their effectiveness to treat infection (4). Use of these antibiotics in lactating animals have variable approvals and tolerances, or maximum residue limits (MRL), in different countries (5–7). In addition, when animal health is a concern, veterinarians may be allowed to use unapproved antibiotics in lactating animals (4). Beta-lactams are the most commonly used antibiotics in milk-producing animals due to their low cost and effectiveness in treating mastitis. Due to this fact, over the last 50 years, milk regulations and detection methods have targeted the detection of the beta-lactam family of compounds (8). Other antibiotic families, such as sulfonamides and tetracyclines, have also been found in milk (9–11). These additional antibiotic families have caused dairy culture failure, or economic damage to milk’s product image, and therefore, are proactively targeted for screening by industry in voluntary testing programs (11). Industry is encouraged to perform their own risk assessment based on drug use and practices and to develop customized screening programs. FDA has provided a risk ranking to aid in this decision process (12). The use of European milk screening tests is less prescriptive and more guidance driven. Therefore, in order to meet dairy stakeholder needs and risk assessments, the industry trend is to use a multiplex rapid test device to target common antibiotics at levels of concern (13–15). This allows the industry to do early proactive on-farm detection of antibiotic residues resulting in reduced volume of milk contamination and avoidance of testing delays at dairies. Less milk disposed of and fewer delays in transport reduces the carbon footprint and is a more sustainable practice. A test that detects beta-lactams, sulfonamides, and tetracyclines, for example, addresses approximately 80% of the antibiotics available for veterinary use (1, 4). Screening methods for bulk tank raw milk are more prescriptively regulated in the United States following procedures developed by the Food and Drug Administration Center for Veterinary Medicine (FDA-CVM). Test manufacturer submitted data are reviewed by FDA-CVM and then, using an independent lab service such as the Association of Official Analytical Chemists Research Institute (AOAC-RI), verified to meet FDA and National Conference of Interstate Milk Shipments (NCIMS) performance specifications. Methods are FDA reviewed, recommended, and then accepted by the NCIMS for bulk tank screening. Bulk tank and tanker testing is done at time of receipt by dairies to control and prevent antibiotic residues in milk (3, 16). The FDA and NCIMS has broadened oversight of screening methods to include other antibiotic families in addition to beta-lactams. NCIMS developed a pilot program for tetracyclines that serves as the regulatory foundation for approving and accepting all raw milk drug residue screening methods (17). This approval process involves the third-party validation of screening tests to confirm performance claims with required detection levels appropriate for detecting tolerance, or target levels, but not overly sensitive to those levels (3). The protocol for the screening test evaluation was developed by FDA-CVM and is available upon request. The protocol is soon to be published in the next, (18th) edition of the Standard Methods for the Examination of Dairy Products. With the completion of the NCIMS pilot program, the FDA wrote a January 2020 Memorandum that encouraged NCIMS to adopt a comprehensive approach to controlling multiple drug residues in milk, including means of verifying the effectiveness of the controls, and consideration of a random multi-drug surveillance program (18). Likewise, in Europe and Canada, the testing trend is to screen for more than beta-lactam antibiotics. Methods for screening milk in Canada need third-party validation of detection levels, performance, and verification to confirm that they are suitable for the intended purpose. The methods then undergo individual provincial review and acceptance procedures coordinated within the Canadian Dairy Commission (19). The objective of this work was to validate a 3 min screening test, a one-step lateral flow test with a single control line, for the simultaneous detection of beta-lactams, sulfonamides, and tetracyclines in commingled raw cows’ milk, and to utilize the data to shepherd the method through the Canadian and U.S. bulk milk tank screening approval processes.

## Materials and Methods

### Test Kit Information

- *Kit Name*.—Charm ROSATRIO test
- *Catalog Number*.—LF-TRIO-20K; LF-TRIO-100K; LF-TRIO-500K
- *Ordering Information*.—Charm Sciences, Inc., 659 Andover St., Lawrence, MA 01843, USA, Tel. 978-687-9200, Fax 978-687-9216 email: info@charm.com, www.charm.com.

### Test Kit Components

- Lateral Flow Test Strips The Charm Rapid One Step Assay (ROSA) TRIO Test (Charm Sciences, Inc. Lawrence, MA) is a lateral flow device that utilizes receptors and antibodies to qualitatively detect beta-lactams, sulfonamides, and tetracyclines at targeted detection levels (20, 21).
- *Positive Control Tablets*.—Containing Penicillin G, Sulfamethazine, and Oxytetracycline to make 5 ppb penicillin G, 10 ppb sulfamethazine, and 100 ppb oxytetracycline when rehydrated with 5mL negative milk.

### Apparatus

- 300 µL pipettor
- EZ reader or manufacturer equivalent capable of reading 4 line lateral flow strip: A common control line is compared to the drug specific lines using an algorithm to analyze, capture, and interpret results (22, 23).
- 56°C ROSA incubator with 3 min timer (when not testing strips with EZ reader incubator)

### Safety Precautions

- Raw milk may contain micro-organisms and hands should be washed after handling.

### General Preparation

- Observe good laboratory practices for microbial testing. Avoid specimen contamination.
- Test on a level surface, in a clean area, and free of dust and blowing air.
- Ambient temperature range should be 10 to 35°C.

### Experimental Design

- The independent laboratory Eurofin-DQCI, Mound View, MN performed tests on spiked drug concentrations and incurred samples; in addition, they prepared blind coded somatic and bacterial interference samples.
- Different fresh commingled raw milk samples obtained from a local farm or dairy, qualified as negative, were used for each drug concentration experiment and for each interference challenge. Drugs for spiking were U.S. Pharmacopeia Convention (USP) traceable with Certificate of Analysis (COA) included.
- The drug stock solutions were prepared and stored per the USP instructions. All reference drug stock solutions were prepared at 1 mg/mL (1000 part per million [ppm], 1 000 000 part per billion [ppb]) concentration, corrected for purity. Prior to use, all raw milk was qualified by M-a-85 approved methods: Charm II Beta-lactam Test Quantitative Assay, Charm II Sulfonamide Test and Charm II Tetracycline Test (17).
- Raw milk was “qualified,” and used for study, if results were within specifications of Charm II Zero Control Standard. The raw milk was also tested (N = 10) with the Charm ROSA Trio Test and each result verified to meet the manufacturer specifications for negative milk.
- The sensitivity evaluation used probability of detection (POD) concentrations determined by FDA-CVM based on concentration response curves submitted by manufacturer. Test strips representing three manufactured lots were integrated into the analysis and evenly distributed between the different concentrations and negative control samples. All samples were randomized and tested as blind samples by at least two analysts over at least two days. Different qualified raw milk samples were used for each drug concentration study.
- Milk naturally contaminated with 1.08 million somatic cells, as determined by Foss somatic cell determination, was obtained from a farm and qualified as antibiotic negative. The milk was split into 5 samples and 4 of those were fortified with antibiotics: penicillin G 3 ppb; cephapirin 18 ppb; sulfamethazine 10 ppb; and oxytetracycline 75 ppb. Stock preparations were divided into 30 replicates. Samples were shipped blind coded to manufacturer for testing. Results were reported to the FDA and AOAC-RI then subsequently sent to the independent laboratory to be decoded.
- Four bacterial isolates were used for milk spiking. Two Gram-negative bacteria were identified to genus level as *Enterobacter* and *Shigella* and two Gram-positive bacteria were identified as *Streptococcus* and *Staphylococcus*. These were cultured in tryptic soy broth overnight at 32°C and added to a qualified, negative raw milk to make three bacterial cocktails (Gram+; Gram–; and Gram± mix) targeting 300 000 CFU/mL using Foss Bactoscan analysis. These samples were split and fortified with antibiotics: penicillin G 3 ppb; sulfamethazine 10 ppb; and oxytetracycline 75 ppb. Twenty split samples of each preparation were blind coded, refrigerated, and sent to the manufacturer overnight for antibiotic testing and aerobic count testing using standard plate agar at 32°C for 48 h. Results were reported to the FDA and AOAC-RI then subsequently sent to the independent laboratory to be decoded.
- Chemical interferences were evaluated with a series of 5 chemical cocktails containing 23 animal drugs unrelated to beta-lactams, sulfonamides, or tetracyclines, and each at the 100 ppb level were added to a qualified, negative raw milk. The five cocktails were: (i) Aminoglycosides: streptomycin, gentamicin, neomycin; (ii) Macrolides, etc.: ivermectin, tilmicosin, erythromycin, novobiocin, pirlimycin; (iii) Thiols, etc.: furosemide, thiabendazole, trichloromethiazide, chlorthiazide; (iv) Hormones: oxytocin, phenylbutazone, dipyrone, dexamethasone; (v) Quinolones, etc.: enrofloxacin, nitrofurone metabolites (AOZ and AMOZ), florfenicol, chloramphenicol, 5-hydroxyflunixin, and para-amino-benzoic acid (PABA). These cocktails were split into four and antibiotics added to three of the splits: penicillin G 3.2 ppb; sulfamethazine 9.7 ppb; and oxytetracycline 86 ppb. These were each tested with the method in triplicate.
- Sensitivity to other chemical analogs in the beta-lactam, sulfonamide, and tetracycline groups, that were not claimed because they are not approved for use in the United States or Canada, were evaluated with the USP drugs made at 100 ppb in qualified raw milk. These were diluted until a concentration was obtained that produced a 90% response on the method.
- Multiple drug detection aspects of the method were investigated with two different experiments. In the first experiment, low levels of each drug causing a less than 50% positive response were tested individually and then combined. This was to demonstrate a theoretical combined cumulative effect of multiple drugs on the method. In a second experiment, very high concentrations of individual drugs, e.g., 10× tolerance (10× MRL for tetracycline), were evaluated to determine if interference to positive drug identification occurred in the other families detected in the same test strip. These samples were blind coded with N = 60 raw milk samples and tested.
- The influence of freezing and thawing milk samples was evaluated with negative and antibiotic fortified milks containing 4 different drugs: penicillin G 3 ppb; cephapirin 18 ppb; sulfamethazine 10 ppb; and oxytetracycline 100 ppb. Milk samples were prepared, tested, and then frozen at −15°C to subsequently thaw, test, and refreeze after 1, 2, 3, 5, and 9 weeks. Separate aliquots with an initial thaw at 3 weeks and a second thaw at 9 weeks, were also evaluated. Samples were slow thawed in cold water before testing. Five replicates from the thawed aliquots were tested each week.
- Incurred beta-lactam samples containing amoxicillin, ampicillin, cephapirin, cloxacillin, or penicillin G were used in the evaluation (20). These were stored at −80°C and had been used in validation of the Charm 3SL3 Beta-lactam Test (21). The same methods of analysis were used to quantitate antibiotics in these thawed incurred samples (24–26).
- Incurred samples of tetracycline were prepared by the University of Iowa following intramammary injection of tetracycline, oxytetracycline, or chlortetracycline drugs into 3 cows with milking 3 times per day. Incurred samples of two sulfonamides, sulfadimethoxine and sulfamethazine, were prepared with inter-uterine bolus administration followed by milking 2 times per day.
- Incurred tetracycline and sulfonamide samples were collected, frozen, and then quantitated using a modification of LC-MS-MS method Lib#4443 (27). Following quantitation, incurred samples for each study were diluted into qualified negative commingled raw cows’ milk to create 5 concentrations: Tolerance or MRL for tetracycline (T/MTL); the 90% positive with 95% confidence (90/95) Level; ½ T/MRL; ¼ T/MRL; and 1/10 T/MRL. Each of these concentrations were then then split into 10 replicates and blind coded with 60 negative raw milk samples. Samples were frozen at −20°C, and sent to the independent laboratory where they were thawed and tested. The manufacturer submitted the blind code to the AOAC-RI prior to testing. After testing the independent laboratory communicated results to the AOAC-RI, FDA-CVM and the manufacturer to decode and report the results.

### TRIO Method Procedure

- Check that incubator temperature is 56 ± 1°C.
- Mix milk sample(s). Place a test strip in incubator, peel back tape of TRIO test strip, exposing sample compartment well and pad.
- Slowly add 300 ± 15 µL milk into the side of the sample compartment well. Reseal tape over sample pad.
- Incubate for 3 min, but <3 min and 30 s. External ROSA incubators have an automatic timer, beeper, and lights to indicate when incubation is completed.
- If using reader incubator, strip will automatically be read at end of incubation period. If using external incubator, remove strips from incubator. Visually verify that the strip has valid development, indicated by a solid and complete control (C) line. Insert valid strip into reader and start analysis, or in case of EZ reader incubate and read mode, the strip is automatically read.

### Interpretation of Results

If any or multiple lines are lighter than the control line, a positive number and positive interpretation specific to the drug family detected are generated: beta-lactam- BL; sulfonamide-S; tetracycline-TE. Multiple drug interpretations are also possible: BLS, BLTE, STE, and BLSTE; see Figure 1.

## Results and Discussion

### Selectivity and Sensitivity Determination

- Table 1 shows the number of positive results for each drug at each concentration (N = 10) by each lot of reagent, as well as the cumulative three lot POD, expressed as % positive. The three different lot number reagents show similar positive responses. The drug study concentrations and POD were used in an XL-Stat program to determine a 90% positive detection level with upper 95% confidence using a probit fit curve of the POD (28). This calculation of sensitivity is consistent with the FDA method of calculating screening test sensitivity which was calculated using a customized SAS program. The calculated independent lab levels as determined by the FDA are reported in the Independent Laboratory column of Table 1. These are compared to manufacturer claim levels that were probit determined from POD data submitted to FDA-CVM (not shown).
- Additionally shown in parentheses in the Manufacturer column of Table 1, are the calculation of CC-β, the EU guidance method to calculate detection capability that produces a concentration with less than 5% negative results using replicate testing results shown in brackets (14, 15). By either method of determining 90% sensitivity with 95% confidence (90/95%), the results of the independent laboratory and manufacturer show agreement (within 20% target/tolerance or MRL) for at least 9 of the 11 studied drugs. The two drugs that were not within 20% were tetracycline and sulfadimethoxine. In both cases the independent lab detected the drugs in raw milk at or below the Target/tolerance or MRL, but the 90/95% levels were 21 ppb less sensitive for tetracycline and 3 ppb less sensitive for sulfadimethoxine compared to the manufacturer claim. In both drug studies the independent lab had 29 positive of 30 replicates at the highest concentration that would add heterogeneity to the 95% confidence calculation causing the higher discrepancy (>20% T/MRL) with the manufacturer data. Overall, results are in agreement with manufacturer, and where there is a discrepancy, the higher levels indicate the claimed drugs are detected lower than T/MRL. There were a total of 2 positive results from the 690 negative samples which is an acceptable error rate less than 0.3%. One of these positive results is believed to be a sample mistake. The other was believed to have been caused by milk flooded over a test line on the strip. This should have been observed as an invalid strip by the operator and repeated without reading. Results of both FDA-CVM and EU-CRL methods for sensitivity determination indicate the method detects the 6 claimed beta-lactams, 2 claimed sulfonamides, and 3 claimed tetracyclines at or below the Canadian MRL and U.S. Target/Tolerances.

**Table 1.** Sensitivity determination of concentration lots of TRIO test. Independent laboratory positive results of concentration different lots N = 10 at each concentration

Drug	Concentration studied (N = 30) except zero (N = 60)	Number positive of N = 10 from each lot except N = 20 for 0			Cumulative probability of detection (POD)^a^ expressed as % positive	Independent lab 90/95% detection level^b^, ppb^c^ (Canadian MRL/U.S. target testing level)	Manufacturer claim 90/95% detection level and (CC-β level),^d^ ppb^c^ [#pos/#tested]
		Lot 006	Lot 009	Lot 010			
Sulfadimethoxine	0	0	0	0	0%	7.6 (10)	4.7(5.0) [40/40]
	1	1	1	2	13%		
	2	3	2	6	37%		
	4	7	7	10	80%		
	6	10	9	9	93%		
	8	10	10	10	100%		
	10	10	9	10	97%		
Sulfamethazine	0	0	0	0	0%	9.2 (10)	7.7(7.7) [59/60]
	2	1	0	0	3%		
	4	0	1	4	17%		
	6	6	8	7	70%		
	8	10	7	10	90%		
	10	10	10	10	100%		
Tetracycline	0	0	1^e^^,^^f^	0	1.6%	42 (100)	21(21) [19/20]
	5	0	0	0	0%		
	10	0	2	1	10%		
	15	4^f^	4	10	60%		
	20	9	10	7	87%		
	100	10	9^i^	10	97%		
Oxytetracycline	0	0	0	0	0%	53 (100)	66(66) [40/40]
	20	0	0	0	0%		
	30	3	7	8	60%		
	40	3	9	8	67%		
	60	10^h^	10	10	100%		
	80	10	10	10	100%		
	100	10	10	10	100%		
Chlortetracycline	0	0	0	0	0%	34 (100)	54(54) [40/40]
	10	2	0	1	10%		
	20	9	6	5	67%		
	40	10	10	10	100%		
	60	10	10	10	100%		
	100	10	10	10	100%		
Penicillin G	0	0	1^e,g^	0	1.6%	2.0 (6)	2.2(2.2) [20/20]
	1.25	3	1	0	13%		
	1.5	8	4	4	53%		
	2	9	10	10	97%		
	2.5	10	10	10	100%		
	5	10	10	10	100%		
Ceftiofur (incurred parent plus metabolites)	0	0	0	0	0%	50.0 (100)	53^j^(53) [40/40]
	40	4	3	3	33%		
	45	8	5	8	70%		
	50	10	10	10	100%		
	60	10	10	10	100%		
	100	10	10	10	100%		
Cloxacillin	0	0	0	0	0%	8.5 (10)	7.4(7.4) [40/40]
	2	0	0	0	0%		
	4	3	0	0	10%		
	6	6	5	7	60%		
	8	9	9	10	93%		
	10	10	10	10	100%		
Amoxicillin	0	0	0	0	0%	3.5 (10)	3.1(3.1) [20/20]
	1.5	2	2	3	23%		
	2	6	5	6	57%		
	2.5	8	6	5	63%		
	3	10	10	10	100%		
	10	10	10	10	100%		
Ampicillin	0	0	0	0	0%	8.8 (10)	7.7(7.7) [55/60] (9.7) [60/60]
	2	0	0	0	0%		
	4	0	0	10	3%		
	6	0	4	8	37%		
	8	8	10	10	93%		
	10	10	10	10	100%		
Cephapirin	0	0	0	0	0%	14.5 (20)	14(14) [38/40]
	4	0	0	0	0%		
	8	0	1	1	7%		
	12	7	8	8	77%		
	16	10	10	10	100%		
	20	10	10	10	100%		

a Cumulative Probability of Detection are used for probit curve generation.

b The upper 95% confidence of the 90% positive level is the 90/95% detection level. These are compared to manufacturer submitted sensitivity calculated by probit and CC-ß concentration (shown in parenthesis) determined from the bracketed [#positive/#tested].

c ppb is parts per billion and is equivalent to µg/kg concentration.

d CC-β is the minimal concentration in which the number of negative results is less than 5% of the total number of tests performed. Shown in parentheses is the CC-β concentration and shown in brackets is [# positive/#tested] at the CC-β concentration.

e There were a total of two positives of 690 negative samples tested < 0.3% positive.

f A Positive-BL observed was likely caused by milk over the BL test line.

g Likely a double test of penicillin G 2.0 ppb sample preceding the zero sample.

h One positive was positive-STE.

i One positive was positive-BLSTE likely caused by milk over BL and S lines.

j Prior claim level was 61 ppb based on synthetic metabolite. This new claim level is based on incurred residue. Parent detection level is about 25 ppb or half of the metabolite level.

### Interferences

- Somatic Interferences*.* There were no positive results in 60 negative samples and no negative results, or wrong drug interpretations, in the 120 positive samples containing 30 replicates each of the 4 antibiotic concentrations; see Table 2. These results indicated no influence by somatic cells at 1.08 million SCC/mL on the method performance.
- Bacterial Interferences*.* There were no positive results from any of the bacterial challenges in 60 negative samples. The penicillin G samples were correctly identified, as positive-BL, 59 of 60 times with 1 negative observation from the penicillin spiked Gram± mix. The sulfamethazine was correctly identified, as positive-S, with all 60 samples. The oxytetracycline was correctly identified 58 of 60 times, as positive-TE, and identified, as positive BLTE, with the other 2 samples both of which were from the Gram– mixture; see Table 2. These results met specifications for concluding there were no bacterial interferences. The bacterial levels in the samples were greater than the targeted Grade A tanker bacterial specification of 300,000 CFU/mL. At time of testing the levels ranged from 700,000 to 13,250,000 CFU/mL. Possible explanations for the actual versus intended bacterial levels include that testing was done 24 h after bacterial spiking and refrigerated shipment, and that the analyses at time of preparation were by flow cytometry versus by standard plate aerobic count at the time of testing. Despite the difference in the intended versus actual bacterial levels in the samples, the conclusion of no bacterial interference was not in doubt. The incidence of the double positives potentially indicated that when the milk became more viscous, the flow characteristics were less optimal and the developments of the control line and/or of the non-spiked drug line were influenced. This risk of a false positive test family result, in the presence of another true positive drug family, supports the need to confirm initial positives with drug family specific tests.
- Chemical Interferences to other animal drugs*.* There were no negative results in the 45 positive samples tested and no positives results in the 15 negative samples tested. There were no interferences from the 5 cocktails evaluated that contained 23 animal drugs; see Table 2.
- Chemical Cross Reactivity to other Antibiotic Analogues. Table 3 lists the drug and concentrations that were estimated to produce 90% positive detection based on reader line intensity. Results demonstrated broad family detection of compounds. In many cases detections were at the relevant MRL of different countries where those drugs are allowed for use.
- Chemical Multi-family cross reactivity. The potential for multiple family cross reactivity was demonstrated with the combined low levels of drugs not expected to give 100% positive: penicillin G 1.25 ppb, sulfamethazine 2 ppb, and oxytetracycline 40 ppb. Individually with N = 10 replicates, the penicillin G 1.25 ppb gave a 70% positive response, sulfamethazine 2 ppb gave a 10% positive response, and oxytetracycline 40 ppb gave a 20% positive response. The combination of these 3 drug concentrations in one cocktail gave a mixture of positive, and dual positive results, totaling a 90% positive response; see Table 2. The resulting 9 positive interpretations from the mixture of drugs were 3 positive-BL; 2 positive-TE; 1 positive-S; 1 positive-BLS; 1 positive-STE; and one positive-BLSTE. While the increase in total positive response is not dramatic, 90% with combined drugs compared to 70% with 1.25 ppb penicillin G response, the experiment demonstrates, in theory, that a cumulative effect of low levels of multiple drugs can combine to produce a stronger combined positive response. In the high drug level evaluation, there were no positive results in the negative samples (0 positives of 60 replicates), and there were no negative results from the positive samples (110 positives of 110 replicates). Additionally, there were no incorrect positive interpretations on the positive group of samples. There were 60 correct positive-BL results (6×N = 10 replicates) from the 6 beta-lactam drugs, 20 correct positive-S results (2×N = 10 replicates) from the two sulfonamide drugs, and 30 correct positive-TE results (3×N = 10 replicates) from the three tetracycline drugs.
- Robustness to freezing. There were no influences from freezing and thawing or from repeated freezing and thawing over the 9 week study period; see Table 2. The robustness of being able to freeze and thaw samples is a requirement of a method in order to conduct incurred residue testing from frozen samples. When a method demonstrates freeze-thaw robustness, incurred samples are able to be prepared from previously frozen samples, then refrozen, and sent to the independent laboratory for evaluation.

**Table 2.** Summary of interference challenges: somatic cells, bacteria, chemical, mixed target drugs, high drug concentrations, and freeze thaw

Influence of Somatic Cells 1.08M SCC/mL						
Negative milk (N = 60)	3 ppb^a^ penicillin G (N = 30)	18 ppb^a^ cephapirin (N = 30)			75 ppb^a^ oxytetracycline (N = 30)	10 ppb^a^ sulfamethazine (N = 30)
0 positive	30 positive BL	30 positive BL			30 positive TE	30 positive S
**Influence of bacteria concentration of bacteria CFU/mL determined by aerobic plate count**						
Bacterial types	Negative milk (N = 20)	3 ppb^a^ penicillin G (N = 20)		75 ppb^a^ oxytetracycline (N = 20)		10 ppb^a^ sulfamethazine (N = 20)
Gram−	0 positive 6 M CFU/mL	20 positive BL 3.1 M CFU/mL		18 positive TE 2 Positive BLTE 3.0 M CFU/mL		20 positive S 9.7 M CFU/mL
Gram+	0 positive 3.9 M CFU/mL	20 positive BL 1.5 M CFU/mL		20 positive TE 0.7 M CFU/mL		20 positive S 8.7 M CFU/mL
Gram−/+ mix	0 positive 13.3 M CFU/mL	19 positive BL 2.0 M CFU/mL		20 positive TE 4.8 M CFU/mL		20 positive S 6.4 M CFU/mL
Total N = 60	0 positive	59 positive		58 positive TE and 2 positive BLTE		60 Positive S
**Influence of animal drug cocktails**						
Chemical group(s)	Negative Milk (N = 3)	3.2 ppb^a^ penicillin G (N = 3)		86 ppb^a^ oxytetracycline (N = 3)		9.7 ppb^a^ sulfamethazine (N = 3)
Aminoglycosides^b^	0 positive	3 positive BL		3 positive TE		3 positive S
Macrolides, etc^c^	0 positive	3 positive BL		3 positive TE		3 positive S
Thiols, etc^d^	0 positive	3 positive BL		3 positive TE		3 positive S
Hormones, etc^e^	0 positive	3 positive BL		3 positive TE		3 positive S
Quinolones, etc^f^	0 positive	3 positive BL		3 positive TE		3 positive S
**Multi-family cross reactivity**						
	1.25 ppb^a^ penicillin G (N = 10)	40 ppb^a^ oxytetracycline (N = 10)		2 ppb^a^ sulfamethazine (N = 10)		1.25 ppb^a^ penicillin G + 40 ppb^a^ oxytetracycline +2 ppb^a^ sulfamethazine (N = 10)
Positive interpretation	7 positive-BL	2 positive-TE		1-positive-S		3 positive-BL,2 positive-TE1 positive-S1 positive-BLS1 positive-STE1 positive-BLSTE
**Influence of high antibiotic concentrations**						
	Negative Milk (N = 60)	beta-lactams (N = 60, 10 each of 6 beta-lactam antibiotics at 10× tolerance)		tetracyclines (N = 30, 10 each of 3 antibiotics at 1000 ppb^a^)		Sulfonamides (N = 20, 10 each of sulfamethazine and sulfadimethoxine at 100 ppb^a^)
Positive Interpretation	0 positives	60 positive-BL		30 positive-TE		20 positive-S
**Frozen and Thawed Milk**						
Weeks	Negative Milk (N = 5)	3 ppb^a^ penicillin G (N = 5)	18 ppb^a^ cephapirin (N = 5)		100 ppb^a^ oxytetracycline (N = 5)	10 ppb^a^ sulfamethazine (N = 5)
1st thaw Week 1	0 positives	5 positive-BL	5 positive-BL		5 positive-TE	5 positive-S
2nd thaw Week 2	0 positives	5 positive-BL	5 positive-BL		5 positive-TE	5 positive-S
3rd thaw Week 3	0 positives	5 positive-BL	5 positive-BL		5 positive-TE	5 positive-S
1st thaw Week 3	0 positives	5 positive-BL	5 positive-BL		5 positive-TE	5 positive-S
4th thaw Week 5	0 positives	5 positive-BL	5 positive-BL		5 positive-TE	5 positive-S
5th thaw Week 9	0 positives	5 positive-BL	5 positive-BL		5 positive-TE	5 positive-S
2nd thaw Week 9	0 positives	5 positive-BL	5 positive-BL		5 positive-TE	5 positive-S

Reported are the number of positive results and the drug positive interpretations: BL = beta-lactam, S = sulfonamide, TE = tetracycline.

a ppb is parts per billion and is equivalent to µg/kg concentration.

b Streptomycin, gentamicin, neomycin.

c Ivermectin, tilmicosin, erythromycin, novobiocin, pirlimycin.

d Furosemide, thiabendazole, trichloromethiazide, chlorthiazide.

e Oxytocin, phenylbutazone, dipyrone, dexamethazone.

f Enrofloxacin, nitrofurone metabolites (AOZ and AMOZ), florfenicol, chloramphenicol, 5-hydroxyflunixin, PABA.

**Table 3.** Cross reactivity to other beta-lactam, sulfonamide, and tetracycline drug analogs

Beta-lactams	Positive detection level, ppb^a^	Sulfonamides	Positive detection level, ppb^a^	Tetracyclines	Positive detection level, ppb^a^
Cefacetrile	30	Sulfabenzamide	200	Doxycycline	250
Cefadroxil	750	Sulfacetamide	35	Minocycline	5000
Cephalexin	750	Sulfachlorpyridazine	1	4-Epi-Tetracycline	750
Cefalonium	10	Sulfadiazine	3	4-Epi-Chlortetracycline	500
Cefazolin	20	Sulfadoxine	20	4-Epi-Oxytetracycline	1000
Cefoperazone	2	Sulfaethoxypyridazine	7	4-Epi-Anhydrotetracycline	1000
Cefquinome	40	Sulfaguanidine	125	4-Epi-Anhydro-chlortetracycline	1500
Cefuroxime	125	Sulfamerazine	4		
Dicloxacillin	10	Sulfamethizole	1		
Nafcillin	100	Sulfamethoxazole	2		
Oxacillin	5	Sulfamethoxypyridazine	5		
Penethamate^b^	2	Sulfanitran	300		
Piperacillin	1	Sulfapyridine	5		
Ticarcillin	25	Sulfaquinoxaline	3		
		Sulfathiazole	1		
		Sulfisoxazole	15		

a ppb is part per billion and is equivalent to µg/kg concentration.

b Penethamate degraded to penicillin in milk within 48 hours and was detected less than 4 ppb.

### Incurred Studies

- *Incurred Beta-lactam Study.* The results of the incurred beta-lactam sample analysis are presented in Table 4. All of the drugs’ T/MRL and 90/95% concentrations were detected 100% of the time indicating the incurred samples are at least as sensitive as spiked samples used in sensitivity determination. Concentrations below than the 90/95% confidence level tended to be less than 100% positive, indicating no hypersensitivity with incurred samples. With amoxicillin and penicillin G only, the next concentration below the 90/95% level was 100% positive; however, two concentrations below produced some negative results. Incurred beta-lactam samples tested on the method were detected similar to, or slightly more sensitive than, fortified samples used in the sensitivity evaluation. Results did not indicate severe hypersensitivity, 100% positive at 1/10 T/MRL, and therefore did not warrant additional warnings in the test method protocol or operating manuals.
- *Incurred Tetracycline Study.* The results of the incurred tetracycline samples are presented in Table 5. OTC at 320 ppb and 60 ppb (the 90/95% level) were detected positive 10 of 10 times (100%), 30 ppb samples were detected positive 9 of 10 times (90%), while 15 and 6 ppb OTC incurred were detected positive 0 of 10 times (0%). CTC at 110 ppb and 70 ppb were detected positive 10 of 10 times (100%), 35 ppb (the 90/95% level) were detected positive 8 of 10 times (80%), 17 ppb CTC incurred were detected positive 4 of 10 times (40%) while 8 ppb were detected 0 of 10 times (0%). TC at 100 ppb and 40 ppb (the 90/95% level) were detected positive 10 of 10 times (100%), 20 ppb were detected positive 2 of 10 times (20%), while 10 ppb and 4 ppb were detected 0 of 10 times (0%). The method detected the three tetracycline incurred residues at about the same concentration levels as were determined for the method using milk fortified with USP standard. The Canadian MRL levels were detected positive 100% of the time as would be expected and the 90/95% levels were detected between 80% and 100% positive. The ½ 90/95% levels ranged from 20% to 90% positive and the ¼ 90/95% levels ranged from 0% to 40% positive indicating the incurred residues behaved similarly to the parent drug determined levels without any indication of hyposensitivity or hypersensitivity to MRL levels. The U.S. tolerance levels (300 ppb total tetracyclines) are greater than MRLs, and therefore, as an initial screening test the method was considered hypersensitive. Confirmation of initial positives should be done with tetracycline specific screening methods to accommodate this hypersensitivity and to address potential cross drug detection errors.
- *Incurred Sulfonamide Study.* The results of the incurred sulfonamide study are shown in Table 6. Sulfadimethoxine (SDM) samples at T/MRL (10 ppb) were detected positive 10 of 10 times (100%). The samples at the 90/95% level (SDM 7.6 ppb) were positive 8 of 10 times (80%). The SDM 3.8 ppb samples, at the ½ 90/95% level, were positive 9 of 10 times (90%). The SDM 1.9 ppb samples, at ¼ 90/95% level, were positive 1 positive of 10 times (10%). The SDM 0.8 ppb samples, at ^1^/_10_ 90/95% level, were positive 0 positive of 10 times (0%). The highest sulfamethazine (SMZ) level attained in a collected milk sample was 8.1 ppb and this was used to represent the target level (10 ppb) and the 90/95% level (9.2 ppb). Sulfamethazine at 8.1 ppb, 4.6 ppb and 2.3 ppb, at the 90/95%, ½ and ^1^/_4_ 90/95% levels, each tested positive 10 of 10 times (100%). The SMZ at 0.9 ppb, at the ^1^/_10_ 90/95% level, tested positive 4 of 10 times (40%). These sulfonamide results indicated a shift in sensitivity of the incurred samples relative to the 90/95% levels calculated from parent drug spiked samples. HPLC-Receptorgram analysis of samples demonstrates some acetyl-sulfonamide metabolites, a liver metabolite (26). These metabolites were contributing to the test response making incurred samples about 50% more positive than the spiked parent sulfonamide compound. Results did not indicate severe hypersensitivity, 100% positive at ^1^/_10_ T/MRL, and therefore did not warrant additional warnings in the test method protocol or operating manuals.

**Table 4.** Incurred beta-lactam drug sample concentrations, number positive and percent positive

Incurred drug	Concentration, ppb^a^	Number positive of N = 10 except zero (N = 60)	% positive
Raw milk	0	0	0%
Penicillin G	0.5	0	0%
	1.25	10	100%
	2^b^	10	100%
	2.5	10	100%
	5	10	100%
Ampicillin	1	0	0%
	2.5	0	0%
	5	6	60%
	9^b^	10	100%
	10	10	100%
Amoxicillin	1	6	60%
	2.5	10	100%
	4^b^	10	100%
	5	10	100%
	10	10	100%
Cloxacillin	1	0	0%
	2.5	0	0%
	5	6	60%
	9^b^	10	100%
	10	10	100%
Cephapirin	2	0	0%
	5	0	0%
	10	6	60%
	15^b^	10	100%
	20	10	100%

a ppb is parts per billion and is equivalent to µg/kg concentration.

b concentrations are closest to the independent laboratory determined 90% detection level with 95% confidence (90/95% level).

**Table 5.** Incurred tetracycline drug sample concentrations, number positive and percent positive

Incurred drug	Concentration, ppb	Number positive of N = 10 except zero (N = 60)	% positive
Raw milk	0	0	0%
Chlortetracycline	8	0	0%
	17	4	40%
	35^a^	8	80%
	70	10	100%
	110	10	100%
Oxytetracycline	6	0	0%
	15	0	0%
	30	9	90%
	60^a^	10	100%
	320	10	100%
Tetracycline	4	0	0%
	10	0	0%
	20	20	20%
	40^a^	10	100%
	100	10	100%

^a^concentrations are closest to the independent laboratory determined 90% detection level with 95% confidence (90/95% level).

**Table 6.** Incurred sulfonamide drug sample concentrations, number positive and percent positive

Incurred drug	Concentration, ppb		Number positive of N = 10 except zero (N = 60)	% positive
Raw Milk	0		0	0%
Sulfadimethoxine	0.8		0	0%
	1.9		1	10%
	3.8		9	90%
	**7.6^a^**		8	80%
	10		10	100%
Sulfamethazine	0.9		4	40%
	2.3		10	100%
	4.6		10	100%
	**8.1** ^a^		10	100%

Bolded concentrations are closest to the independent laboratory determined 90% detection level with 95% confidence (90/95% level).

a The greatest incurred sample concentration contained 8.1 ppb which is less than the 90/95% level and the target (T/MRL) levels of 10 ppb.

### Additional Manufacturer Data (Robustness, Stability, QC Manufacture)

- Manufacturer tested the robustness of assay steps using a Youden model multivariate experiment testing high and low parameters of milk temperature 0 or 7°C, milk volume ±5%, incubation time 3 and 4 min, incubation temperature ±1°C, time to read result at 30 s and 3 min, time to start the assay at 30 s and 90 s, and ambient temperature 10 and 35°C. Raw negative commingled milk was fortified with penicillin G 3.2 ppb, cephapirin 16.8 ppb, sulfamethazine 9.7 ppb, and oxytetracycline 86 ppb. Samples were tested N = 3 times in 8 different assay configurations to isolate N = 12 parameter specific readings. There were no positive errors in negative samples and no negative errors in the positive samples. Probability of reader differences are reported in Table 7 and results less than 0.01 and 0.05 are considered significant. Assay incubation was the most significant and at 4 min enhanced more positively the positive test readings, particularly the beta-lactams, but did not have an effect on negative samples. Other parameter tests having to do with time, if they did have an effect, tended to enhance more positively the readings of the β-lactam drugs. Generally, ambient temperature 10 to 35°C, milk volume variation ± 5%, milk temperature 0 to 7°C, 3 additional minutes to read result, 90 s to start the assay, and milk fat 0 to 5.5% had minimal effect on assay performance; see Table 7.
- Charm TRIO tests are quality tested after manufacture following the Charm Sciences, Inc. quality control specifications. Manufacturer provided evidence lot to lot quality performance using a scientific random sampling program and a series of performance specifications appropriate to raw commingled milk sample testing. Performance at end of shelf life, at 12 months refrigerated, was verified with real time storage samples and accelerated stress testing. This testing is to verify kit performance and that sensitivity does not shift from initial manufacture.

**Table 7.** Multi-variate robustness experiments—probability values from paired T test of reader values N = 12

Assay parameter	Perturbation	Raw milk	3.2 ppb Pen G	16.8 ppb Cephapirin	9.7 ppb SMZ	86 ppb OT
Milk Temperature	Control 4°C					
	0°C	0.892	0.613	0.457	0.757	0.831
	7°C	0.111	0.800	0.460	0.404	0.575
Milk volume	Control 300 µL					
	285 µL	0.738	0.573	0.189	0.627	0.256
	315 µL	0.130	0.453	0.347	0.790	0.941
Incubation time	Control 3 min					
	4 min	0.401	0.008^a^	0.000^a^	*0.011*	*0.025*
	4 min	0.941	0.186	0.000^a^	0.750	0.166
Time of reading	Control < 1 min					
after test	3 min	0.365	*0.041*	0.142	0.187	0.009^a^
completed	3 min	0.101	0.057	*0.026*	0.010^a^	*0.024*
Ambient	RT 20–22°C					
temperature	10°C	0.010^a^	*0.013*	0.066	0.005^a^	*0.018*
	35°C	0.690	0.846	0.472	0.710	0.732
Incubation	Control 56°C					
temperature	55°C	0.074	0.421	0.870	0.540	0.462
	57°C	0.379	0.372	0.992	0.239	*0.046*
Assay set-up	Control < 15 s					
time	30 s	0.112	0.264	0.120	0.348	0.178
	90 s	0.360	0.005^a^	0.142	0.140	0.299

a P < 0.01 and italicized are P < 0.05.

## Conclusions

The TRIO Test method was the first simultaneous beta-lactam, sulfonamide, and tetracycline multiplex lateral flow antibiotic detection test validated and accepted in both the United States and Canada for screening raw commingled cows’ milk. This validated method is an additional tool for quality testing and verifying residue control programs in the dairy industry. Such testing and programs ensure the high purity of milk and maintain consumer confidence. To meet the requirements for screening milk in the United States, the method was evaluated by NCIMS on a drug family basis and accepted two separate times for beta-lactams and sulfonamides, and a third time the method was accepted for tetracycline screening through the tetracycline pilot program. In December 2018, the method approvals were incorporated into FDA memorandum M-a-85 Rev. 16 (16). While beta-lactam and sulfonamide regulatory limits in the U.S. and Canada are similar, the tetracycline levels differ. The MRL of Canada (and the EU) is 100 ppb total tetracyclines including their metabolites, while the U.S. tolerance is 300 ppb total tetracyclines including their metabolites. In Canada the method was adopted among the provinces where positive samples are followed up with farm investigation and LC-MS confirmation. In the United States, the method was adopted by NCIMS as a screening method for all three drug families to be confirmed positive using family specific tests that target the U.S. tolerance/target levels (17). This U.S. confirmation procedure addressed the differences in tetracycline regulatory limits, allowing a more sensitive initial screen to meet MRL levels, while confirming initial positives at U.S. regulatory tolerances. The specific drug confirmation also addressed the rare instance where a milk constituent anomaly, or a multi-drug mixture, might cause a positive screening result that could be determined negative at legal thresholds with drug family specific testing. The screening method detected three of the most common antibiotic families used in animal health treatment and meets most countries regulatory requirements. Multi-drug screening methods, such as this one, may be a useful tool in verifying the control of antibiotics in animal health within the dairy industry. The method attributes, particularly the screen for tetracyclines at MRL and confirmation at tolerance, promote international trade and raw material quality standards for purchases acceptable to dairy stakeholders including producers, processors and regulatory agencies. The regulatory acceptance of this multiplex method in the United States and Canada provides assurances that the method is robust and suitable for use in raw milk procurement practices. This rapid, simple test format provides dairy stakeholders additional sustainable tools for comprehensively addressing antibiotic residues determined probable from risk assessments.

### Submitting Company

Charm Sciences, Inc. 659 Andover St, Lawrence, MA 01843

Independent Laboratory

Eurofins-DQCI, 5205 Quincy Street, Mounds View, MN 55112

### Reviewers

**Joe Boison**

Canadian Food Inspection Agency

Saskatoon Laboratory—Centre for Veterinary Drug Residues

116 Veterinary Road

Saskatoon Saskatchewan Canada S7N 2R3

**Philip Kijak**

U.S. Food and Drug Administration, Center for Veterinary Medicine

Office of Research—Director Division of Residue Chemistry

8401 Muirkirk Rd. Laurel, MD 20708

**Jim Agin**

Q Laboratories, Inc.

1400 Harrison Avenue

Cincinnati, Ohio 45214-1606
